# Red biases sex categorization of human bodies

**DOI:** 10.3389/fpsyg.2023.1234417

**Published:** 2023-09-07

**Authors:** Na Chen, Koyo Nakamura, Katsumi Watanabe

**Affiliations:** ^1^The Gonda Multidisciplinary Brain Research Center, Bar-Ilan University, Ramat Gan, Israel; ^2^Department of Cognition, Emotion, and Methods in Psychology, Faculty of Psychology, University of Vienna, Vienna, Austria; ^3^Japan Society for the Promotion of Science, Tokyo, Japan; ^4^Faculty of Science and Engineering, Waseda University, Tokyo, Japan

**Keywords:** red, sex categorization, color-gender association, body, background color

## Abstract

Color is associated with gender information (e.g., red-female). However, little has been known on the effect of color on sex recognition of human bodies. This study aimed to investigate whether the color red could influence the categorization of human bodies by sex, and the effect of contextual information. Visual stimuli were created using body silhouettes varying along the waist-to-hip ratio from female to male shapes. These stimuli were presented in conjunction with red, green, and gray colors, which were used either as body color (Experiment 1) or background color (Experiment 2). Participants were instructed to categorize the sex of the body stimuli as either male or female by pressing labeled keys. The results showed that when red was used as a body color, it induced a bias toward feminine body perception, while when used as a background color, it induced a bias toward masculine body perception. Thus, the color red influenced the sex categorization of human bodies, which being modulated by contextual information. These findings provided novel insights into the effect of contextual color cues in sex recognition of human bodies.

## 1. Introduction

Recognizing the sex of an individual from a distance, particularly when facial or body features are not easily distinguishable, often relies on various cues. Colors are sometimes used as a means of recognition. For instance, the color red could be stereotypically associated with women based on their clothing colors. In visual media, positioning a character against a red background, especially in intense scenes like walking away from an explosion, can evoke perceptions of heroism or villainy, as seen in Hollywood action movies like Wolverine and the X-Men (2011), which are often associated with male characters. Those differing effects of perceiving red highlight the importance of context in sex recognition. In addition to physical information from the visual body itself, color serves as the fastest and most direct visual cue for sex recognition, playing a crucial role in quickly assessing potential rivals or mates (Nestor and Tarr, 2008). Despite its potential significance, to the best of our knowledge, no previous study has examined the effect of color on the categorization of sex in human bodies. Specifically, the effect of contextual information, when applied directly to the body or present in the background, on this sex process remains unexplored.

Color carries gender information in both Western and Eastern countries. In Western societies, pink has been stereotypically associated with girls and blue with boys since the early 20th century (Pomerleau et al., 1990; Cunningham and Macrae, 2011). There is a prevalent tendency for individuals to have different color preferences for their children based on gender. They tend to choose pink colors for their female children, including pink toys, clothing, and room decorations, while choosing blue or green colors for male children (Del Giudice, 2017; Endendijk, 2022). These gender-stereotyped color perceptions in early life may lead to color-gender associations. In Japan, gender-stereotyped colors are frequently used in social and public service systems. For instance, female toilet icons are typically colored in red or pink, while male toilet icons are colored in blue, green, or black. Previous studies have shown that Japanese people associate reddish colors with females and bluish/black colors with males (Kitagami et al., 2009, 2010; Ishii et al., 2019; Mochizuki and Ota, 2022; Chen et al., 2023a,b). It is common for reddish colors to be widely used in clothing, cosmetics, and activities for females, while blue/green colors are more frequently used in male clothing and behaviors/interests in modern daily life. The mere exposure to the co-occurrence of color and gender information during social interactions may contribute to the establishment of color-gender associations (e.g., red-female). This color-gender association plays a significant role in shaping people’s gender-related cognition and behavior. In a recent study by Chen et al. (2023b), the red-female association could be automatically activated and influenced both conceptual gender categorization and perceptual font color discrimination through Stroop-word categorization tasks. It is possible that when the color red was applied to human bodies, it may evoke the learned red-female associations based on the mere exposure to co-occurrences of red clothing and female body perception.

Meanwhile, it should be noted that red has also been associated with masculine traits, such as dominance, aggression, danger, anger, and testosterone levels (Stephen et al., 2012; Young et al., 2013; Wiedemann et al., 2015; Jonauskaite et al., 2021a; Chen et al., under review). People can use the presence and intensity of red facial color information when categorizing the sex of faces, as male faces are typically redder and darker than female faces (Van den Berghe and Frost, 1986; Yip and Sinha, 2002; Frost, 2006; Nestor and Tarr, 2008; Carrito and Semin, 2019). Thus, in certain contexts, red can also be associated with males. For instance, when the color red is present in the background of an ambiguous human body, it may evoke impressions of dominance, aggression, and danger, potentially leading to the perception of a male body. According to the color-in-context theory proposed by Elliot and colleagues, colors acquire specific meanings through environmental and social interactions, while also being influenced by evolutionary factors (Elliot and Maier, 2014). Importantly, the influence of color is contingent on psychological contexts. For instance, the effects of the color red on approach or avoidance behaviors can vary depending on the context. In a romance-related context, red may elicit approach behavior, whereas in a competition-related context, it may lead to avoidance behavior (Meier et al., 2012).

The categorization of human bodies based on sex is strongly influenced by their visual appearance, including features such as the waist-to-hip ratio (WHR) and shoulder-to-hip ratio (Johnson et al., 2012; Gandolfo and Downing, 2020). Individuals with a lower WHR are more likely to be identified as females rather than males (Pazhoohi and Liddle, 2012). Additionally, social contextual information also plays a role in sex categorization. Studies have shown that a fear condition can enhance a bias toward perceiving bodies as male (Johnson et al., 2012), while the presence of a child next to a target can lead to a higher likelihood of attributing female sex (Brielmann et al., 2015). It has been proposed that sex categorization involves a multimodal process, governed by both perception and high-level cognition processes, including stereotypes (Freeman and Ambady, 2011; Clifford et al., 2015). In other words, the categorization process is shaped by both low-level processes that involve visual features and higher-level cognitions formed through prior experiences. Given these findings, it is plausible that the sex categorization of human bodies could be influenced by various factors, including color-gender associations.

**Figure 1:**
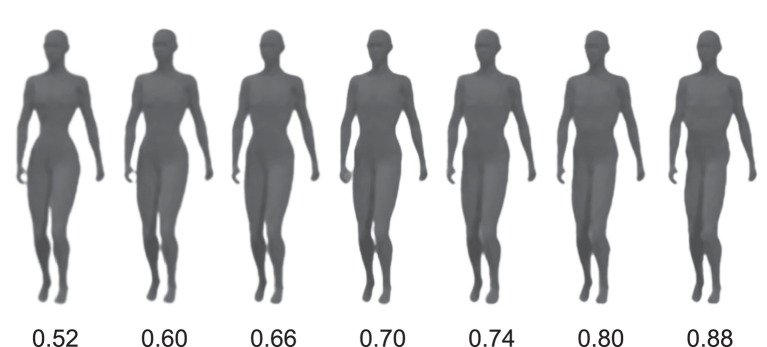
Body stimuli varying in seven levels of waist-to-hip ratio (WHR).

Based on the previous findings, the current study aimed to investigate the effect of the color red on the categorization of human body by sex, in combination with contextual effects related to both body color and background color. Through two experiments, we manipulated the perceived masculinity and femininity of body shapes, incorporating the contextual information of red color. This information was used both when the color was applied as the actual body color and when it served as a background color. In the first experiment, we examined whether a red body color could lead to a bias in the sex categorization of human bodies, comparing it to green and gray body colors. In the subsequent experiment, we investigated how contextual color cues might modulate the impact of color on body-sex perception. This was achieved by presenting the body stimuli against different background colors, namely red, green, and gray. The body stimuli were adapted from Johnson et al. (2012) and encompassed a range of waist-to-hip ratios (WHR) representing variations in perceptions of feminine and masculine bodies (see Figure 1). The colors were selected based on their gender associations. Green, for instance, is commonly linked with male, and it’s situated opposite to red in many well-established color models (Fehrman and Fehrman, 2004). We did not select blue as a choice due to its prevalence as a more preferred color among both males and females (Jonauskaite et al., 2019), as well as its lack of gender bias in previous research (Li et al., 2021; Jonauskaite et al., 2021a). Gray, an achromatic color, was chosen as a contrasting color that could be standardized for saturation and lightness (Elliot and Niesta, 2008; Young et al., 2013). Participants were required to categorize the gender of body stimuli presented with the three different body colors in the first experiment or against the three different background colors in the second experiment. We hypothesized that a red body color would lead to a bias toward perceiving the body as female, while a red background would instead induce a bias toward perceiving the body as male.

## 2. Experiment 1

### 2.1. Methods

#### 2.1.1. Participants

Twenty-nine Japanese undergraduate students (17 males, mean age = 20.2 years, *SD* = 1.8) from Waseda University participated in the experiment. All participants had normal or corrected-to-normal visual acuity and normal color vision, and were naïve regarding the purpose of the experiment. The sample size was set *a priori* at 23 participants, based on a target of 0.8 power with a medium effect size by power analysis (Cohen’s *d* = 0.6). We collected more data in case of some participants’ data could not fit the psychometric functions (e.g., Deviation >100). This experiment, as well as the subsequent experiment, was approved by the institutional review board (IRB) of Waseda University (2015-033), and conducted in accordance with the ethical standards of the 1964 Declaration of Helsinki. Written informed consent was obtained from all participants in advance.

#### 2.1.2. Apparatus and stimuli

E-Prime 2.0 (Psychology Software Tools, Inc.) was used to present the stimuli and collect the data. Stimuli were displayed on a 24-inch LCD monitor (EIZO FG2421, EIZO corp, Hakusan, Japan), with a 1920 × 1080-pixel resolution and a refresh rate of 100 Hz. Participants viewed the monitor binocularly at a distance of approximately 60 cm.

**Figure 2:**
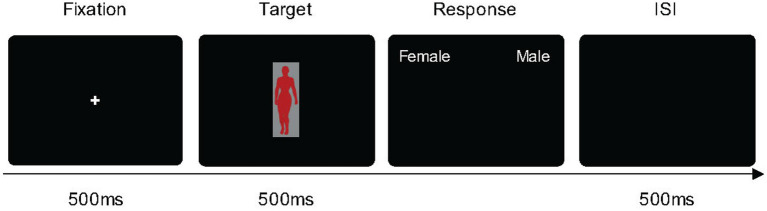
The paradigm of Experiment 1. Each trial began with a fixation cross for 500 ms, followed by a colored-body target for 500 ms, and participants pressed the key to report the sex judgment of the body.

Seven computer-generated bodies that varied in a sexually dimorphic cue [WHR = 0.52, 0.6, 0.66, 0.7, 0.74, 0.8, 0.88 from the body stimuli in Johnson et al. (2012) and Figure 1] were used. The body stimuli were fitted in a frame of 135 × 380 pixels with gray background color (with 57.37/1.37/255.96 in the CIE LCh color space), presented in three different body colors created by Photoshop CS2 (Adobe Systems Inc., San Jose, CA, United States). The body stimuli were presented against a black screen background throughout the experiment (see Figure 2). The three colors used in the present study were measured by PR-655 (Photo Research, Chatsworth, CA, USA). The color information was as follows: Red: L* = 56.66, a* = 80.59, b* = 57.60; Green: L* = 60.03, a* = −64.56, b* = 41.64; Gray: L* = 57.59, a* = −2.46, b* = −30.15.

#### 2.1.3. Procedure

The experiment was conducted in a laboratory under dim lighting conditions. At the beginning of the experiment, participants were instructed to denote the sex of a centrally presented body shape stimulus by pressing a key on the computer keyboard (i.e., pressing ‘*z*’ for a female body using the left index finger or ‘*m*’ for a male body using the right index finger). Each trial began with a fixation for 500 ms, followed by a body stimulus in body colors (i.e., red, blue, or gray) for 500 ms, after which a black screen was presented until the participants responded (see Figure 2). They were requested to identify the sex of the body stimulus (male or female) as quickly as possible. Trials were separated by an inter-stimulus interval (ISI) of 500 ms. Each body stimulus was presented 15 times in a random order, resulting in 315 trials (7 bodies × 3 body colors × 15 repetitions = 315 trials). The experiment was preceded by ten practice trials. At the end of every 35 trials, participants took a self-determined break. The entire experiment took approximately 20 min to finish.

### 2.2. Analysis

All statistical analyses were performed using R (R Core Team, 2020). For each participant and condition of body color (i.e., red, green, and gray), we determined a psychometric function based on the proportion of trials in which the body was judged as female. That is, each visual stimulus was repeated 15 times, the proportion of responses with judging as female was calculated. The sex categorization rate from each participant was fitted with a psychometric function using a generalized linear model, with a binomial distribution. By fitting a psychometric function, the level of stimulus variable at which perceptual performance transitions from one perceptual category to the other [i.e., the point of subjective equality (PSE)], and the precision with which participant is able to perceptually differentiate stimuli along the dimension [i.e., the just noticeable difference (JND)] can be revealed (Klein, 2001). To examine the effect of body color on body-sex categorization, the PSE and JND obtained from the fitted curves were used as indicators of perceived differences in sex judgments and discrimination sensitivity, respectively. The PSE was taken from the *x* value at which the fitted curve had a *y* value of 0.5, i.e., the WHR of the body stimuli being judged switching from “female” to “male” (where the body looked equally male and female). Thus, the PSE is the WHR value denoting the maximum uncertainty of sex information. The JND was calculated from one-half of the difference between the x values at which the fitted curve had y values of 0.50 and 0.75. This is the amount of change necessary in a body stimulus for the change to be detected 50% of the time. When the JND value is small, a participant is better able to discriminate the sex information. The PSEs and JNDs of the three body colors from the fitted curves of each participant were compared using Repeated measure ANOVA and paired-sample *t*-test with Bonferroni’s correction for multiple comparisons (*α* = 0.05/3 = 0.017). The Bayes Factors (BF10) were further referred to determine whether or not there was support in favor of the alternative (H1) or null (H0) hypotheses (Morey and Rouder, 2018). A value of 1 means that null and alternative are equally likely, larger values suggest that the data are in favor of the alternative hypothesis, and smaller values indicate that the data are in favor of the null hypothesis.

To verify the consistency and robustness of the results, the categorical responses were also fitted by means of Generalized Linear Mixed Model (GLMM) with the *glmer* function (Jaeger, 2008; Bates et al., 2015). We included all fixed and random effects as a first instance and then to eliminate those factors that reduce the model’s overall goodness of fit (Bolker, 2008). The Akaike information criterion (AIC) was also used to compare the goodness of fit of the alternative models (a lower AIC value indicating a higher quality model; Mazerolle, 2019). After the model selection, analysis of variance (Type II Wald *x*^2^ test) was performed to identify significant effects and interactions, and Tukey *post hoc* pairwise comparisons were also performed. All data and code are available online.^1^

### 2.3. Results

The data of five participants which failed to fit the psychometric function were removed from the data analysis (the deviances of the fitted models of the excluded participants were all above 120, and the mean deviance of the included participants was 80.11; see the individual plots-Exp1 in the online repository (see footnote 1). Data from 24 participants were used for the data analysis.

**Figure 3:**
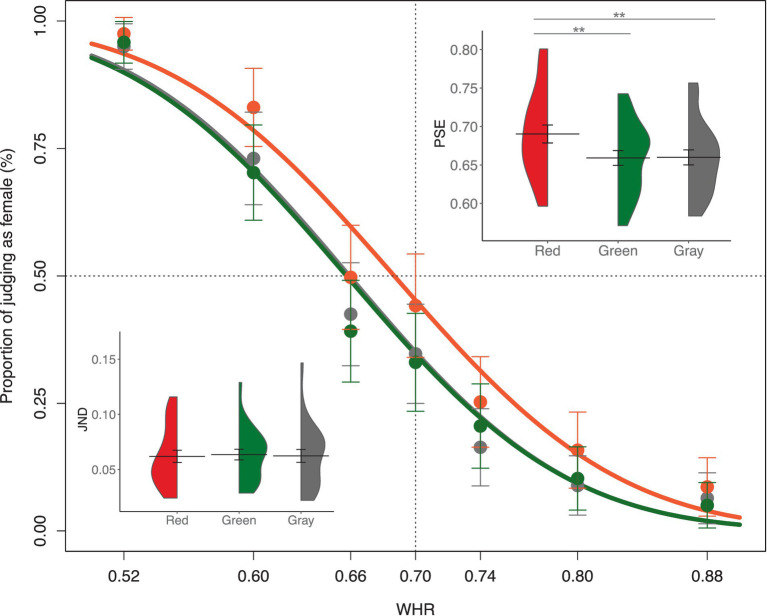
The female proportion for each body stimulus. The mean PSEs and JNDs for the three body color conditions are presented. Error bars represent the standard mean error. Asterisks indicate significant differences (**p < 0.01, Bonferroni corrected).

For the total amount of male and female responses, irrespective of the WHR manipulation, participants made male responses more frequently than female responses (*t*(23) = 3.32, *p* = 0.003, Cohen’s *d* = 0.68). The mean proportion of female responses to each body stimulus and their psychometric curves for the three body colors are shown in Figure 3. The data points represent the mean proportion of trials in which participants responded with female judgment for each body stimulus in the three body colors. The psychometric function showed a horizontal left shift for all the three body colors, indicating that participants’ sex judgment of the body stimuli was biased toward a male perception. Repeated measure ANOVA showed that there are significant difference on PSEs between the three body colors, *F*(2, 46) = 13.72, *p* < 0.01, *η*_p_^2^ = 0.08. The PSEs in red body color (Mean = 0.69, *SD* = 0.06) were significantly larger than those in gray body color (Mean = 0.66, *SD* = 0.05), *t*(23) = 3.86, Bonferroni corrected *p* = 0.002, Cohen’s *d* = 0.79, BF_10_ = 42.56, and in green body color (Mean = 0.66, *SD* = 0.05), *t*(23) = 4.76, Bonferroni corrected *p* = 0.0003, Cohen’s *d* = 0.97, BF_10_ = 316.73. No difference was observed in PSEs between gray and green background colors, *t*(23) = 0.15, Bonferroni corrected *p* = 1, Cohen’s *d* = 0.03, BF_10_ = 0.22. Thus, red body color biased the sex categorization of bodies toward a female body perception, reduced the general shift toward a male body bias. Repeated measure ANOVA analysis of JNDs showed that there was no significant difference between the three body colors, *F*(2, 46) = 0.13, *p* = 0.88, *η*_p_^2^ = 0.01. Thus, there was little effect of body color on the sensitivity of sex perception of human bodies.

As a further test of population categorical responses, these responses were fitted using a GLMM. We anticipated that the sex judgment of body would depend on those two factors with the body color and the WHRs. The generalized linear mixed-effect model analysis showed that there was no significant interaction effect between the body color and WHRs, *x*(12)^2^ = 8.85, *p* = 0.72. After removing the interaction effect from model analysis, a final model including the body color and WHRs as fixed factors (AIC = 6143.93) showed a significant main effect of body color, *x*(2)^2^ = 62.31, *p* < 0.001, and WHRs, *x*(6)^2^ = 1790.08, *p* < 0.001. Post-hoc multiple comparison showed a significant difference on the logit scale between the red and gray body colors (Tukey’s HSD, *p* = 0.00001), and between the red and green body colors (Tukey’s HSD, *p* = 0.00001), and no difference between the gray and green body colors (Tukey’s HSD, *p* = 0.92). Thus, these analyses performed on the categorical responses led to the same conclusion: the red body color was more likely to bias body stimulus toward a feminine body than green and gray body colors.

In summary, this experiment demonstrated that the presence of a red body color led to a bias in sex categorization toward a female body perception, compared with green and gray body colors. These findings suggest that the red body color might trigger an automatic red–female association, influencing the sex categorization process. In the next experiment, we investigated whether the contextual information of color cues could alter the observed effect of red-female association on body sex categorization. This was accomplished by comparing the sex categorization responses for bodies presented against three background colors: red, green, and gray.

## 3. Experiment 2

### 3.1. Methods

#### 3.1.1. Participants

Twenty-five newly recruited Japanese undergraduate students (11 males, mean age = 20.3 years, *SD* = 1.1) from Waseda University participated in Experiment 2. All participants had normal or corrected-to-normal visual acuity and normal color vision and were naïve regarding the purpose of the experiment.

#### 3.1.2. Stimuli and procedure

**Figure 4:**
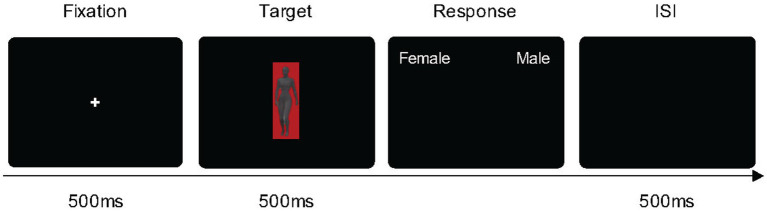
The paradigm of Experiment 2. Each trial began with a fixation cross for 500 ms, followed by a target body in a colored-background for 500 ms, and then participants pressed the key to report the sex judgment of the body.

The body silhouettes were identical to those used in Experiment 1. The visual stimuli were body silhouettes in gray color [set as gray (57.37/1.37/255.96) in the CIE LCh color space] and presented in the three background colors (i.e., the same color value of red, green, and gray as used in Exp. 1; Figure 4). The experimental setting and procedure were identical to those of Experiment 1.

### 3.2. Analysis

The data analysis was identical to that in Experiment 1.

### 3.3. Results

One participant whose response data failed to fit the psychometric function were removed from the data analysis (the deviance of the fitted models of the excluded participant was above 100, and the mean deviance of the fitted models of the included participants was 75.6; see the individual plots-Exp2 in the online repository (see footnote 1).

**Figure 5:**
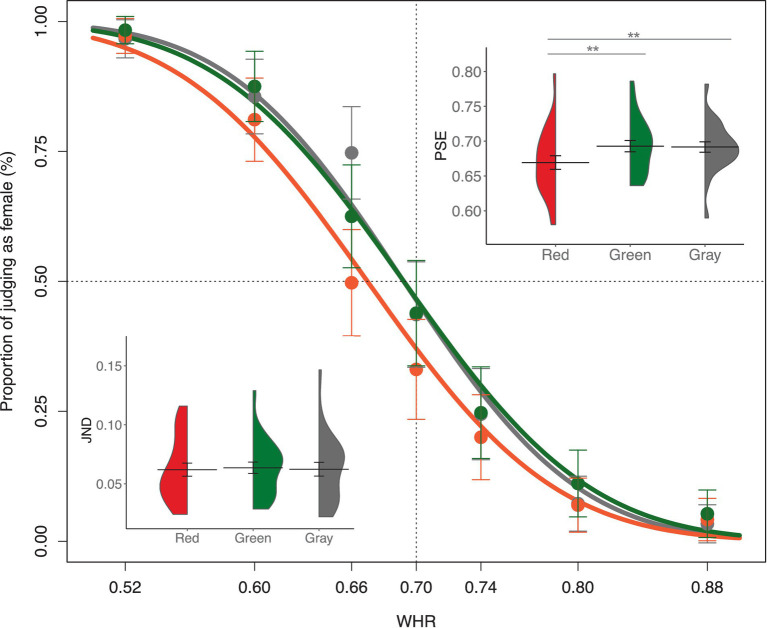
The female proportion for each body stimulus. The mean PSEs and JNDs for the three background color conditions are presented. Error bars represent the standard mean error. Asterisks indicate significant differences (**p < 0.01, Bonferroni corrected).

For the total amount of male and female responses, participants made male responses more frequently than female responses (*t*(23) = 2.18, *p* = 0.04, Cohen’s *d* = 0.45). The proportion of female response to each body stimulus and their psychometric curves in the three background colors are shown in Figure 5. Repeated measure ANOVA showed that there are significant difference on PSEs between the three background colors, *F*(2, 46) = 9.83, *p* < 0.01, *η*_p_^2^ = 0.07. The PSEs in red background color (Mean = 0.67, *SD* = 0.05) were significantly smaller than in gray background color (Mean = 0.69, *SD* = 0.04; *t*(23) = 3.78, Bonferroni corrected *p* = 0.003, Cohen’s *d* = 0.77, BF_10_ = 36.40), and in green background color (Mean = 0.69, *SD* = 0.04; *t*(23) = 3.38, Bonferroni corrected *p* = 0.008, Cohen’s *d* = 0.69, BF_10_ = 15.28). No difference was observed in PSEs between the gray and green background colors (*t*(23) = 0.22, Bonferroni corrected *p* = 1, Cohen’s *d* = 0.05, BF_10_ = 0.22). Thus, a red background color could bias the body-sex toward a male body perception, compared with gray and green background colors. Repeated measure ANOVA analysis of JNDs showed no significant difference between the three background colors, *F*(2, 46) = 0.87, *p* = 0.43, *η*_p_^2^ = 0.01, suggesting that the sensitivity of body-sex perception may not be influenced by the background colors.

A final model including the interaction and main fixed factors (AIC = 5780.36) showed a significant interaction effect between the background color and WHRs, *x*(12)^2^ = 33.41, *p* = 0.0008, a significant main effect of background color, *x*(2)^2^ = 49.48, *p* < 0.001, and a significant main effect of WHRs, *x*(6)^2^ = 1955.58, *p* < 0.001. Post-hoc multiple comparison showed a significant difference on the logit scale between the red and gray background colors (Tukey’s HSD, *p* = 0.026), and between the red and green background colors (Tukey’s HSD, *p* = 0.0001), and no difference between the gray and green background colors (Tukey’s HSD, *p* = 0.23). Thus, categorical responses also showed that the red background color was more likely to bias the body stimulus toward a masculine body than green and gray background colors.

This experiment showed that a red background color could bias sex categorization of bodies toward a male body perception. As background color is a task-irrelevant visual cue, it may influence body-sex categorization in different levels of visual and cognition processing, compared with the body color that on main body figures (Minami et al., 2018; Peromaa and Olkkonen, 2019). Thus, the contextual information also plays a role in red-sex bias on human bodies.

## 4. Discussion

This study is the first to provide evidence that red color can serve as an effective cue for the sex categorization of human bodies. Specifically, our findings revealed that the contextual information influenced the effect of red on body-sex categorization, as the presence of red cues had distinct effects when presented as either the body color or the background color. Through two experiments, we demonstrated that an ambiguous body depicted in red color induced a bias toward perceiving it as female, whereas when the body was present against a red background, it induced a bias toward perceiving it as male. These results contribute to filling the research gap by revealing the effect of color as a contextual information on the sex categorization of human bodies.

One intriguing aspect of the effect of red on body-sex categorization is the opposite effect between the two conditions, where red used as a body color induces a female body bias, while red used as a background color induces a male body bias. One possible explanation for this result may be that the visual processing of sex for human bodies involves multimodal interactions, including interactions between bottom-up perceptual cues and top-down stereotype effects. Previous research has highlighted the dynamic interactive processes in this context, emphasizing the interplay between sensory information, prior knowledge, and attentional factors (Freeman et al., 2008; Freeman and Ambady, 2011; Clifford et al., 2015; Jonauskaite et al., 2021b). According to the dynamic interactive model, bottom-up processing and top-down attention jointly constrain the activation of sex categories through an ongoing interactive process. During the processing of the sex of human body, the sensory information of body features (e.g., WHR) and color cues interact with stored knowledge, represented as the prior probability distribution, leading to a decision of sex categorization.

When red was used as the body color, the interaction between the stored knowledge of red-female association and the sexually dimorphic visual cue of the waist-to-hip ratio appeared to contribute to the formation of a cohesive perception of body sex. This interaction might have activated top-down effects, such as stereotypes related to the association between red body and feminine characteristics, potentially being automatically triggered. It is worth noting that, in many cultural contexts, females tend to wear red clothing more frequently compared to males (Frank, 1990). Consequently, the activation of the red-female association, combined with the impact of expectations and memories, could have influenced the processing of visual cues and led to a bias toward perceiving the bodies as feminine. These findings align with previous research demonstrating the influence of red-female associations on perceptions and categorizations (Cunningham and Macrae, 2011; Chen et al., 2023a,b). For instance, Cunningham and Macrae (2011) showed that a pink *t*-shirt increased perception of femininity, while a blue *t*-shirt increased perception of masculinity.

When red was used as a background color, it induced a bias toward perceiving the bodies as masculine, contradicting the feminine bias observed with red body color. This suggests that the contextual information plays a significant role in shaping the impact of red on the processing of body-sex. The background color, as a contextual task-irrelevant visual cues, interacting with body silhouettes, contributed to the visual configuration in body-sex processing. Plenty of research has found that the color red is associated with visual cues related to testosterone, dominance, aggression, threat, anger, and danger, all of which are associated with masculine characteristics (Elliot and Niesta, 2008; Elliot and Maier, 2012, 2014; Fetterman et al., 2012; Stephen et al., 2012; Young et al., 2013; Pravossoudovitch et al., 2014; Wiedemann et al., 2015; Mentzel et al., 2017; Ruba et al., 2021). In many non-human animals, the presence of red on a conspecific is interpreted as a threat cue, signaling dominance, aggression, and fighting ability (Pryke, 2009; Khan et al., 2011). In humans, testosterone surges during aggressive/dominance encounters can cause visible reddening of the face (Montoya et al., 2005; Stephen et al., 2012), and the experience of anger also leads to reddening of the skin (Changizi et al., 2006). Consequently, red colors carry significant biological signals, upon which impressions of masculinity may be built. In a previous study, Chen et al. (under review) observed that red background color enhanced the perception of dominance in human faces. Facial dominance and masculinity are positively correlated (i.e., the more masculine a face appears, the more dominant it is perceived; Hill et al., 2013). Thus, a red background color might evoke associated impressions of masculinity (e.g., dominance/aggression/anger/danger) through a specific cognitive processing pathway, leading to a bias toward perceiving the bodies as male (Schneider, 2005; Stephen et al., 2012; Young et al., 2013; Wiedemann et al., 2015).

The contextual information provided by color cues influences the recognition of the sex of human bodies, with body color and background color leading to different patterns of sex recognition. A red background color may trigger a biological masculine association, whereas red body color may induce learned red-female associations in a top-down manner. When red is applied to the body interacting with features such as waist-to-hip ratio (WHR), it may reduce the masculine bias and potentially trigger red-love-feminine associations. Numerous studies have shown that red is associated with love, romance, and sexual attractiveness (Elliot et al., 2013; Jonauskaite et al., 2020a; Pazda et al., 2021). For instance, red roses and hearts symbolize love on Valentine’s Day, and red lingerie signifies romance in literature. Those results suggest that color-gender interactions are context-dependent and highly dynamic, processed at different levels of cognition.

One limitation of the current study is that we only used three colors (red, green, and gray) to examine the effect of red on sex categorization. Gray was used as a control color; however, it might also be associated with masculinity to some extent. Defining a gender-neutral color can be challenging. Future research may explore the gender associations linked with each individual color, such as natural skin colors (e.g., yellow, brown, black, white), to reveal the strongest and least associated colors for female and male. Another limitation is that each visual stimulus was repeated 15 times, which may have resulted in a response frequency bias, such as participants made male responses more frequently than female responses. Future studies should use an even number of repetitions to avoid response bias. Moreover, future studies are needed to explore the mechanism underlying the effect of color on body-sex perception by examining the effect of perceptual color dimensions (e.g., hue/lightness/chroma; Dael et al., 2016), top-down processing (e.g., color–sex associations/color semantic/color emotion; Jonauskaite et al., 2020b), and their interactional effects.

In conclusion, our findings highlight the role of red as contextual information in the sex categorization of human bodies. We demonstrated that participants encoded specific patterns of color information related to bodies, with red color playing a role at different levels of body-sex processing. Specifically, when used as a body color, red induced a bias toward female body, whereas when used as a background color, red induced a bias toward male body. These findings provide novel insights into the effect of color as contextual information on the processing of sex-related information in human bodies.

## Data availability statement

The raw data supporting the conclusions of this article will be made available by the authors, without undue reservation.

## Ethics statement

The studies involving humans were approved by the Institutional Review Board (IRB) of Waseda University (2015-033). The studies were conducted in accordance with the local legislation and institutional requirements. The participants provided their written informed consent to participate in this study.

## Author contributions

NC coded the experimental paradigm, collected and analyzed the data, and drafted the manuscript. KN and KW provided critical revisions. All authors contributed to the study design and approved the final version of the manuscript for submission.

## Funding

This work was supported by Grant-in-Aid for Scientific Research (grand numbers: 19K20387, 20K22296, 21K13759, 22K13801, and 22H00090) from Japan Society for the Promotion of Science.

## Conflict of interest

The authors declare that the research was conducted in the absence of any commercial or financial relationships that could be construed as a potential conflict of interest.

## Publisher’s note

All claims expressed in this article are solely those of the authors and do not necessarily represent those of their affiliated organizations, or those of the publisher, the editors and the reviewers. Any product that may be evaluated in this article, or claim that may be made by its manufacturer, is not guaranteed or endorsed by the publisher.

## Footnotes

^1^ https://osf.io/djygf/
